# Osteonecrosis of the distal tibia in systemic lupus erythematosus: A rare case report

**DOI:** 10.1016/j.ijscr.2020.10.069

**Published:** 2020-10-28

**Authors:** Ihsan Oesman, Danarto Hari Adhimukti

**Affiliations:** Department of Orthopaedics and Traumatology, Faculty of Medicine, Universitas Indonesia, Cipto Mangunkusumo Hospital, Jakarta, Indonesia

**Keywords:** osteonecrosis, distal tibia, systemic lupus erythematosus

## Abstract

**Introduction:**

Osteonecrosis (ON) is characterized by cellular death of bone components due to interruption of blood supply that leads to bone ischemia and potential joint destruction. There are multiple risk factors and medical condition associated with ON, including systemic lupus erythematosus (SLE). The most common sites of ON are the femoral head, distal femur, proximal humerus, talus and lumbar spine. Very few cases of nontraumatic ON in distal tibia have been reported in the literature.

**Case Illustration:**

We present a case of 23-year-old female diagnosed with osteonecrosis of distal tibia and history of SLE. The patient also had history of avascular necrosis of right hip and underwent right total hip arthroplasty. We treated the patients with conservative treatment for intial management.

**Discussion:**

The risk of ON in SLE patients is likely due to the results of both the SLE itself and use of corticosteroids. Systemic inflammation in SLE reduces the development of osteoblasts, increases osteoclast maturation and activity and increases protohrombotic agents that can lead to rapid bone loss. Corticosteroids are the most consistent risk factor associated with the development of ON in SLE. Conservative medical management is effective in the early stages of the disease before bone collapse.

**Conclusion:**

Despite advances in the diagnosis and treatment of SLE, symptomatic ON continues to be a significant comorbidity. Strategies to detect and manage early stage ON is necessary to prevent the progression of this serious complication.

## Highlights

• Despite advances in the diagnosis and treatment of systemic lupus erythematosus (SLE), symptomatic osteonecrosis continues to be a significant comorbidity.
• Strategies to detect and manage early stages of osteonecrosis is necessary to prevent progression of this serious complication.
• We present a case of 23-year-old female diagnosed with osteonecrosis of the distal tibia and history of SLE.

## 1 Introduction

Osteonecrosis (ON), also known as avascular necrosis of the bone, is characterized by cellular death of bone components due to interruption of blood supply that leads to bone ischemia and potential joint destruction. The exact mechanism of ON is still unclear, some mechanisms involved include ischaemia, vascular occlusion, intraosseous microcirculation coagulation, and mechanical stress [1]. There are multiple risk factors and medical condition associated with ON, including trauma, alcohol abuse, smoking, vascular disease, renal disease, coagulation disorders, and rheumatic diseases, especially in systemic lupus erythematosus (SLE) [1,2].

Systemic lupus erythematosus (SLE) is a chronic, multisystem autoimmune disease with unknown aetiology that predominantly affects women. Several factors have been associated with the development of osteonecrosis in SLE but the most consistent association is corticosteroid (CS) therapy [3]. Osteonecrosis occurs mainly in SLE patients who are treated with corticosteroids and is extremely rare among SLE patients who had never received corticosteroids [4]. The reported prevalence of symptomatic osteonecrosis in SLE is 4%–15% and up to 40% in asymptomatic patients [5]. Osteonecrosis most often affects young adults aged 30–50 years. MRI is the gold standard diagnostic method to detect both symptomatic and asymptomatic ON [6].

The most common sites of ON are the femoral head, distal femur, proximal humerus, talus and lumbar spine [6]. X-rays, MRIs, and scintigraphy are helpful methods for detecting lesions from osteonecrosis. ON in multiple sites is rare, being reported in 3.3% of patients with ON. Multiple osteonecrosis in patients with SLE is also unusual. The term multifocal osteonecrosis is used to describe the presence of osteonecrotic lesions in three or more anatomical sites [1]. Few case reports have described ON affecting the ankle, and the talus is the more common site of injury than the distal tibia [2]. Very few cases of nontraumatic ON in distal tibia have been reported in the literature. In this study, we present a case report of patient with osteonecrosis of distal tibia due to history of SLE. This paper has been written according to the SCARE 2018 statement [7].

## 2 Case report

A 23-year-old female presented with worsening pain in the left ankle in the last 4 weeks before admission. Patient previously treated by internist with complaints of pain and swelling in the left ankle in the last 2 months. Pain did not decrease with pain killers and were accompanied by redness of the ankles, warmness and difficulties to move. There was no history of trauma and fever. The patient was given antibiotics and the pain was reduced but still felt intermittently.

The patient was diagnosed with SLE with kidney involvement 3 years ago. A kidney biopsy was performed with result of lupus nephritis. Patient consumed methylprednisolone 2 × 4 mg every 2 days. Two years ago, patient complained of right pelvic pain with difficulty in walking and diagnosed with avascular necrosis due to SLE. Right total hip arthroplasty was done at Cipto Mangunkusumo Hospital.

From the physical examination, the left ankle is swollen and hyperemic. There is no open wound or discharge. Plantar flexion-dorsoflexion is limited due to pain (5–20 degree). Distal sensory and motoric are within normal limit.

The laboratory result showed an increased leukocyte, C-Reactive Protein, and D-dimer. Others components were within normal limit. From radiological examination, there was multiple lytic lesions in the distal epi-meta-diaphysis of the left tibia, accompanied by a solid type periosteal reaction on the postero-medial side of the metaphysis on the side of the left distal tibia. MRI of left ankle showed multifocal appearance of osteonecrosis in the distal epimethaphysis of the tibia, talus, calcaneus and left first shaft metatarsal.

## 3 Discussion

Osteonecrosis can be classified into post-traumatic and nontraumatic. Post-traumatic osteonecrosis usually caused by traumatic displacement of bone fragments, which leads to impaired blood supply and ischemia to the affected bone. On the other hand, nontraumatic osteonecrosis associated with variety of systemic diseases and clinical conditions [8]. In this study, we present a case of a patient with osteonecrosis of distal tibia without history of trauma. Patient has been suffered from systemic lupus erythematosus since 3 years ago with no other disease. Among the systemic diseases, osteonecrosis is strongly associated with SLE. Osteonecrosis is one of the serious complications for SLE [9].

The pathophysiology of ON is not completely understood. It is believed to be result of the combined effects of metabolic factors, genetic predisposition, and several factors affecting blood supply [10]. Mechanisms that have been proposed for nontraumatic ON include increased intraosseous pressure, arterial emboli or thrombosis, venous occlusion, and coagulation disorder [11]. The risk of ON in SLE patients is likely the result of both the SLE disease state itself and the use of corticosteroids as medication [8]. Incidence of ON in SLE patients was affected by disease activity, the higher disease activity score is significantly associated with accelerated incidence of ON, while lower disease indicates comparable safe status for ON [9]. The overall effect of glucocorticoids on bone is multifactorial.

There are some proposed potential mechanisms. Systemic inflammation in SLE reduces the development of osteoblasts (by producing oxidized LDL), causes apoptosis of osteoblast and increases osteoclast maturation and activity (by increasing TNF levels). Increased osteoclast maturation and reduced osteoblast maturation/ activity make rapid bone loss can occur [6]. Systemic inflammation also increases protohrombotic agents (homocysteine), which might induce thrombosis of small blood vessels could lead to cellular necrosis of a number of osteocytes. This necrosis might be followed by localized demineralization of the bone and fractures can occur in this weakened bone [11]. In our case, systemic inflammation marked by elevated leukocyte and C-reactive protein.

Corticosteroids (CS) are the most common cause of non-traumatic ON as well as the most consistent factor associated with the development of ON in SLE. Although the effect of CS on ON seems to be clearly established, the pathophysiology is not fully understood.1 The effect of corticosteroids at the cellular level and their influence on the immune system is one of the principally proposed mechanisms for the development of ON [1]. Glucocorticoid receptors have been found in cartilage, osteoblasts, osteoclasts, and osteocytes. Binding of glucocorticoids to these receptors has been shown to induce an anti-inflammatory response through apoptotic pathways within the immunogenic cells. Thus, osteoclasts and osteoblasts can undergo apoptosis after prolonged treatment with glucocorticoids [2]. Apoptosis of the osteoblasts and osteoclasts, a decreased bone turnover and a decreased survival of osteocytes appear to be important mechanisms for inducing ON [1]. It also has been hypothesized that chronic CS use promotes intraosseous adipocyte hypertrophy and deposition of fat within the intramedullary tissue, which causes elevation of intracortical pressure and compromises perfusion. Another postulated mechanism is that CS alter lipid metabolism, leading to fat microemboli in subchondral vessels [10]. The duration of corticosteroid therapy, total cumulative dose and highest daily dose have been independently associated with the development of ON.4 The median duration of glucocorticosteroid use prior to the development of osteonecrosis was 3.4 years, showing that a short exposure of CS may be associated with osteonecrosis [5].

The clinical presentation of ON can be manifest as asymptomatic or present with gradual-onset pain that can progress to severe pain, bone collapse, and joint damage. These conditions can lead to restriction of the range of motion involved joints [10]. Bilateral hip involvement occurs in up to 90% of SLE patients with ON. The most commonly affected other joints include the knee and shoulder, with incidence of 10%–20%. Less commonly, the ankle and elbow can be involved [11]. Multifocal ON affecting more than three of these joints is not infrequent in SLE patients, with approximately 3% incidence [8]. In this study, our patient already underwent right total hip arthroplasty due to AVN and began suffering pain in the left ankle with swollen and hyperemic soft tissues.

Diagnosis of osteonecrosis in SLE can be made by radiography findings but always begins with a thorough assessment for common risk factors associated with ON and physical examination. Radiographic evaluation should begin with standard radiographs of all symptomatic joints. In addition, patients with ON should have their femoral heads evaluate although symptoms occurred in other joints because of the high incidence of femoral head involvement (greater than 80%) in multifocal disease [11]. The most commonly used radiographic staging method is the Ficat and Arlet system, which was originally applied to the femoral head but can be applied to any involved bone. Plain radiographs can assess for evidence of articular-surface collapse. Magnetic resonance imaging (MRI) is the gold standard and can be used for diagnosing early-stage ON lesions (sensitivity and specificity > 99%) [8]. From MRI examination, our patient has multifocal appearance of osteonecrosis in the distal epimethaphysis of the tibia, talus, calcaneus and left first shaft metatarsal. To the best of our knowledge, this is a very rare case.

Conservative medical management is the first line of nontraumatic ON treatment, which is most effective in the early stages of the disease before bone collapse. The medical management approach is to reduce systemic inflammation and use of glucocorticoids, as both are risk factors for osteonecrosis, and to improve the vasculature to the affected bone area [6]. The conservative management of ON included pharmacological treatment, modification of activity with restricted weight bearing, and the used of ultrasound or electric signals to stimulates repair. Medications that have been investigated in several studies and have been used in patients with ON included bisphosphonates (inhibition of osteoclast activity), statins (pro-osteoblastic and anti-adipogenic effects on bone marrow stromal cells), heparin (stabilization the balance between the fibrinolytic and thrombotic cascades), antimalarials (antithrombotic and lipid-lowering properties) and supplementation with folic acid (reduce homocysteine levels) [2,10].

In general, joint-preserving procedures are the first line of operative treatment for pre-collapse lesions. Several operative treatments in the early stages for nontraumatic ON of the ankle included core decompression, vascularized and nonvascularized bone grafting, tibiotalar fusion, and talectomy with tibiocalcaneal fusion. These procedures have been used as a treatment method to relieve intraosseus pressure, increase blood flow to the necrotic subchondral bone, and in some cases provide structural support for the underlying subchondral bone. In end-stage of nontraumatic ON of ankle, when subchondral collapse has occurred, it usually leads to ankle arthrodesis [2,8].

## 4 Conclusion

Although ON is often secondary to trauma, there are some other etiologies including SLE. Despite advancements in the diagnosis and treatment of SLE, symptomatic osteonecrosis continues to be a significant comorbidity. Strategies to detect and manage early stages of ON is necessary to prevent progression of this serious complication.

## Declaration of competing interest

None.

## Funding

None.

## Ethical approval

Not required.

## Consent

Written informed consent was obtained from the patient for publication of this case report and accompanying images. A copy of the written consent is available for review by the Editor-in-Chief of this journal on request.

## Author contribution

Ihsan Oesman: performing the procedure, data collection.

Danarto Hari Adhimukti: performing the procedure, data collection, writing the paper.

## Registration of research studies

Not required.

## Guarantor

Ihsan Oesman.

## Provenance and peer review

Not commissioned, externally peer-reviewed.
